# Supplementary material for: Genomic Amplification of UBQLN4 Is a Prognostic and Treatment Resistance Factor
Source: Cells. 2022 Oct 21;11(20):3311. doi: 10.3390/cells11203311 (PMC9600423; doi:10.3390/cells11203311)
Supplement: Supplementary file 1 [file cells-11-03311-s001.zip › cells-1948079-supplementary.pdf]

Genomic Amplification of *UBQLN4* Is a Prognostic and Treatment Resistance Factor

Yuta Kobayashi [1], Matias A. Bustos [1], Yoshiaki Shoji [1], Ron D. Jachimowicz [2,3,4,5], Yosef Shiloh [6] and Dave S. B. Hoon [1,*]

Supplementary Materials

Supplementary Figures

Figure S1. Comparison of *UBQLN1-4* mRNA expression fold-change between tumor and normal/adjacent normal tissues in different cancers.

Figure S2. Survival analysis according to *UBQLN4* mRNA levels in Stage III/IV patients.

Figure S3. Genomic alterations and methylation status of *UBQLN4* gene.

Figure S4. Post-transcriptional regulation of *UBQLN4* mRNA levels.

Figure S5. Distributions of DNA copy number of the *UBQLN1-3* genes in different solid tumors in the TCGA database.

Figure S6. Copy number alterations in adjacent normal tissues in TCGA dataset

Figure S7. *UBQLN4* mRNA levels in relation to AJCC stage and histopathology subtypes in different cancer types.

Figure S8. *UBQLN4* mRNA levels in relation to AJCC stage in different cancer types.

Figure S9. *UBQLN4* DNA copy number in relation to AJCC stage and histopathology subtypes in different cancer types.

Figure S10. Survival analysis according to *UBQLN4* DNA copy number in Stage I/II patients.

Figure S11. Associations between *UBQLN4* mRNA levels and Cisplatin and Olaparib responses in *BRCA1/2* wild-type ovarian cancer cell lines.

Supplementary Table

Table S1. Summary of *UBQLN4* analysis using The Cancer Genome Atlas (TCGA) and The

Genotype-Tissue Expression (GTEx)

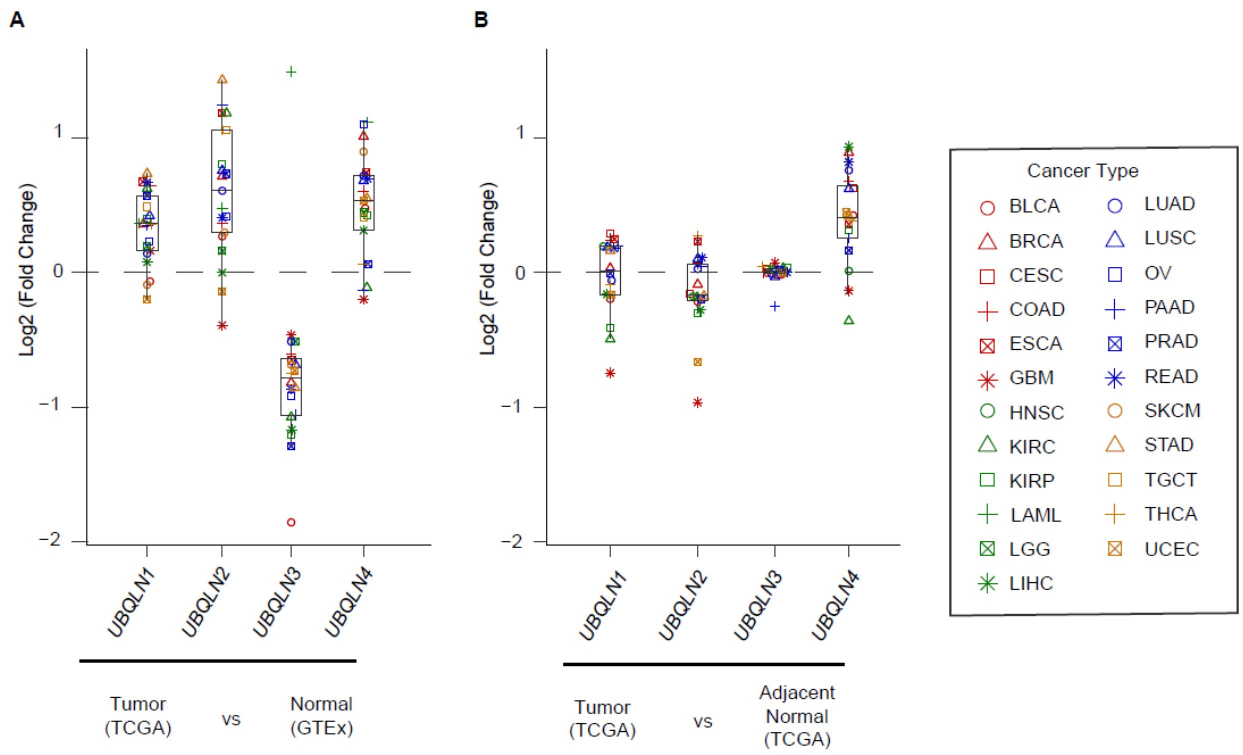

Figure S1. Comparison of *UBQLN1-4* mRNA expression fold-change between tumor and normal/adjacent normal tissues in different cancers

A. Comparison of mRNA levels between tumor (TCGA) and normal tissues (GTEx). **B.** Comparison of mRNA levels between tumor (TCGA) and adjacent normal tissues (TCGA). Each cancer type was represented with a symbol as indicated in the Figure.

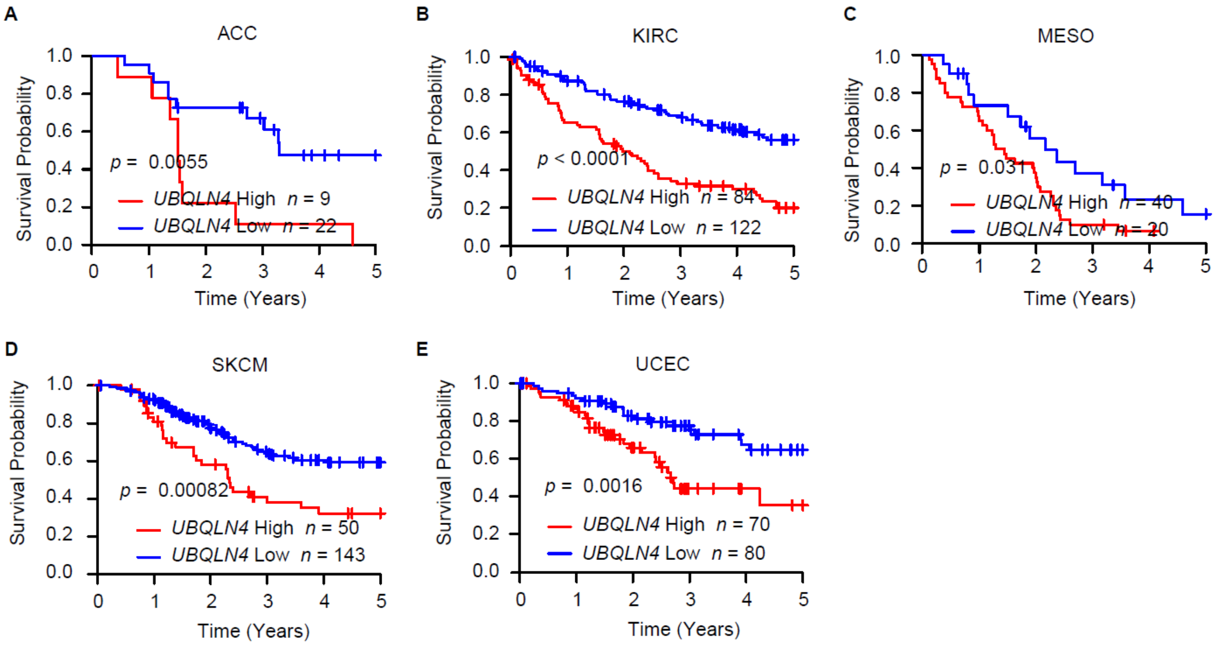

Figure S2. Survival analysis according to *UBQLN4* mRNA levels in Stage III/IV patients

A-E. Kaplan-Meier curves for ACC, KIRC, MESO, SKCM, and UCEC patients according to *UBQLN4* mRNA expression in Stage III/IV using TCGA datasets. ACC: Adrenocortical Carcinoma, KIRC: Kidney Renal Clear Cell Carcinoma, MESO: Mesothelioma, SKCM: Skin Cutaneous Melanoma, UCEC: Uterine Corpus Endometrial Carcinoma.

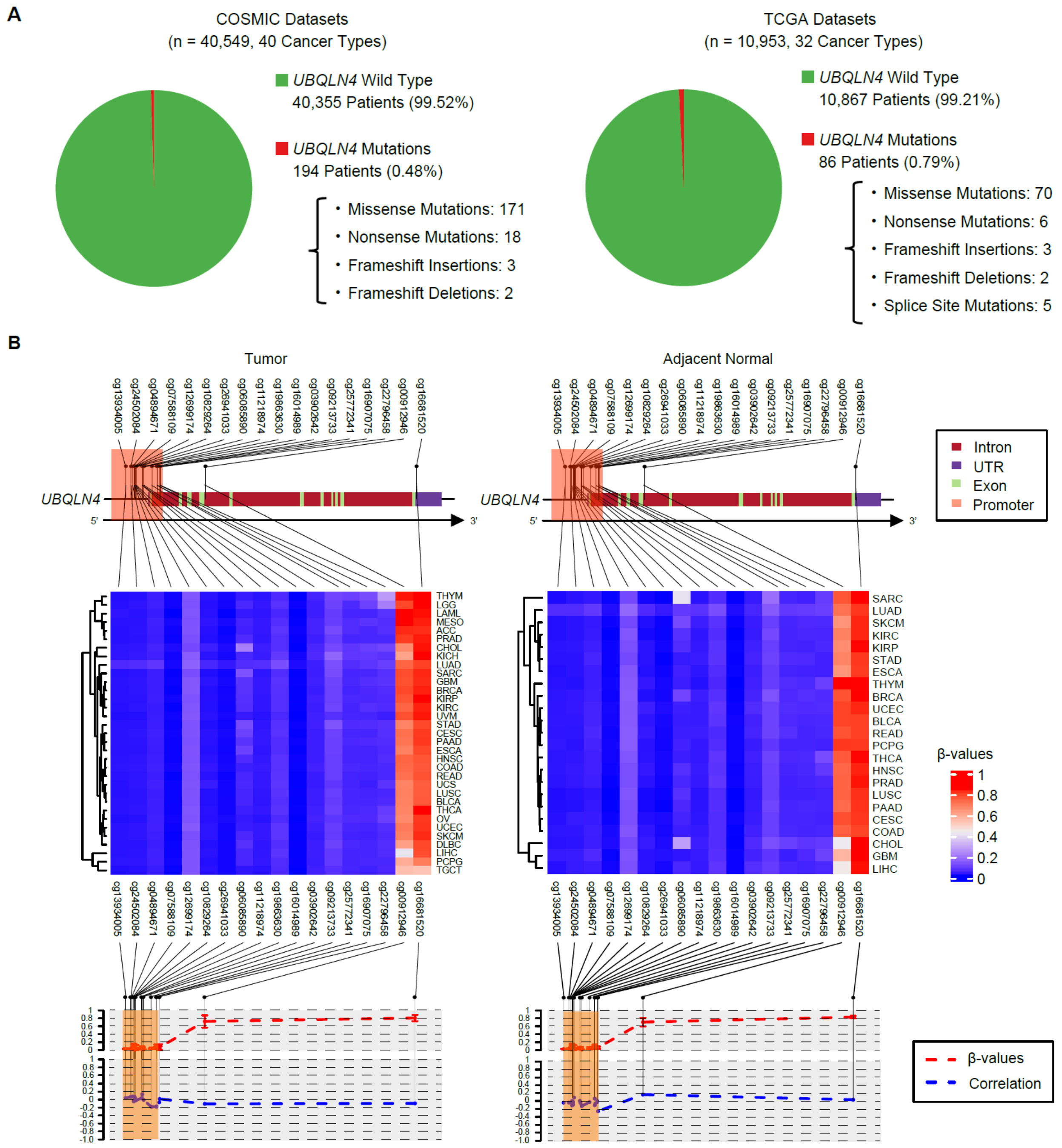

Figure S3. Genomic alterations and methylation status of *UBQLN4* gene

A. Pie chart showing the percentage of *UBQLN4* mutations in pan-cancer cases of the Catalogue of Somatic Mutations in Cancer (COSMIC) (Left, *n* = 40,355) and TCGA (Right, *n* = 10,953) databases. **B.** DNA methylation status of *UBQLN4* gene in tumor tissues (Left) and adjacent normal tissues (Right) in the TCGA pan-cancer datasets. (Top) *UBQLN4* gene structure, promoter regions, and methylation probe sites in HM450K methylation dataset. (Middle) Mean beta values at each probe site in each cancer type were shown

as a heatmap. (Bottom) The mean beta values (red dotted line) and Pearson's correlation (blue dotted line) between beta values for each probe and *UBQLN4* mRNA levels in pan-cancer were calculated.

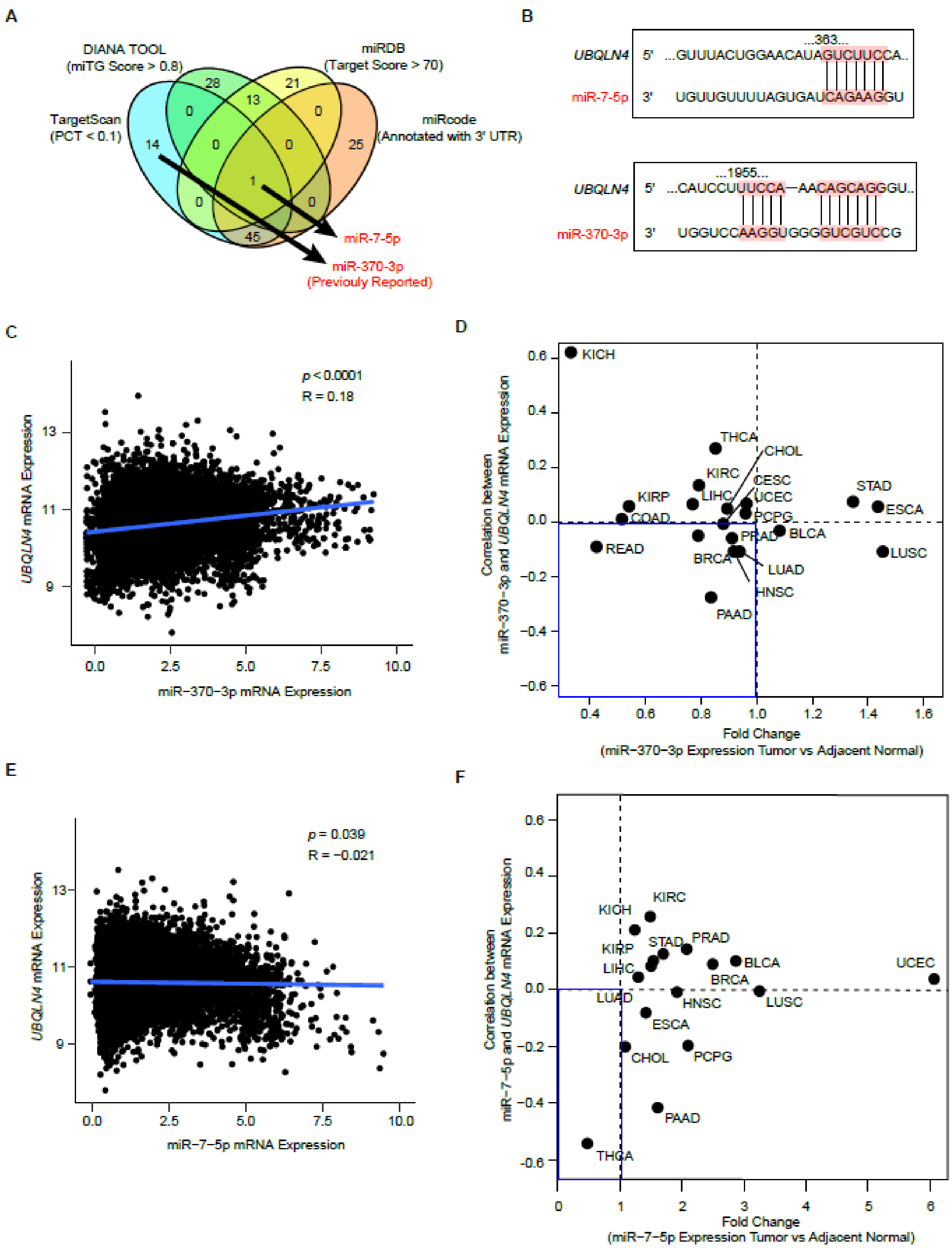

Figure S4. Post-transcriptional regulation of *UBQLN4* mRNA levels

A. Venn diagram showing putative miRs that target *UBQLN4* mRNA predicted by four different computational tools (TargetScan, miRCODE, DIANA TOOL, and miRDB). **B.** miR-370-3p and miR-7-5p

sequences aligned with *UBQLN4* sequences. **C.** Pearson's correlation between miR-370-3p levels and *UBQLN4* mRNA levels in pan-cancer in TCGA datasets. **D.** Statistical evaluation of miR-370-3p in TCGA datasets. Each cancer type is represented as a dot. The horizontal coordinate indicates the fold change in miR-370-3p levels in tumor tissues compared to adjacent normal tissues. The vertical coordinate indicates the Pearson correlation coefficient between miR-370-3p and *UBQLN4* mRNA levels. **E.** Pearson's correlation between miR-7-5p levels and *UBQLN4* mRNA levels in pan-cancer in TCGA datasets. **F.** Statistical evaluation of miR-7-5p in TCGA datasets. Each cancer type is represented as a dot. The horizontal coordinate indicates the fold change in miR-7-5p levels in tumor tissues compared to adjacent normal tissues. The vertical coordinate indicates the Pearson correlation coefficient between miR-7-5p and *UBQLN4* mRNA levels.

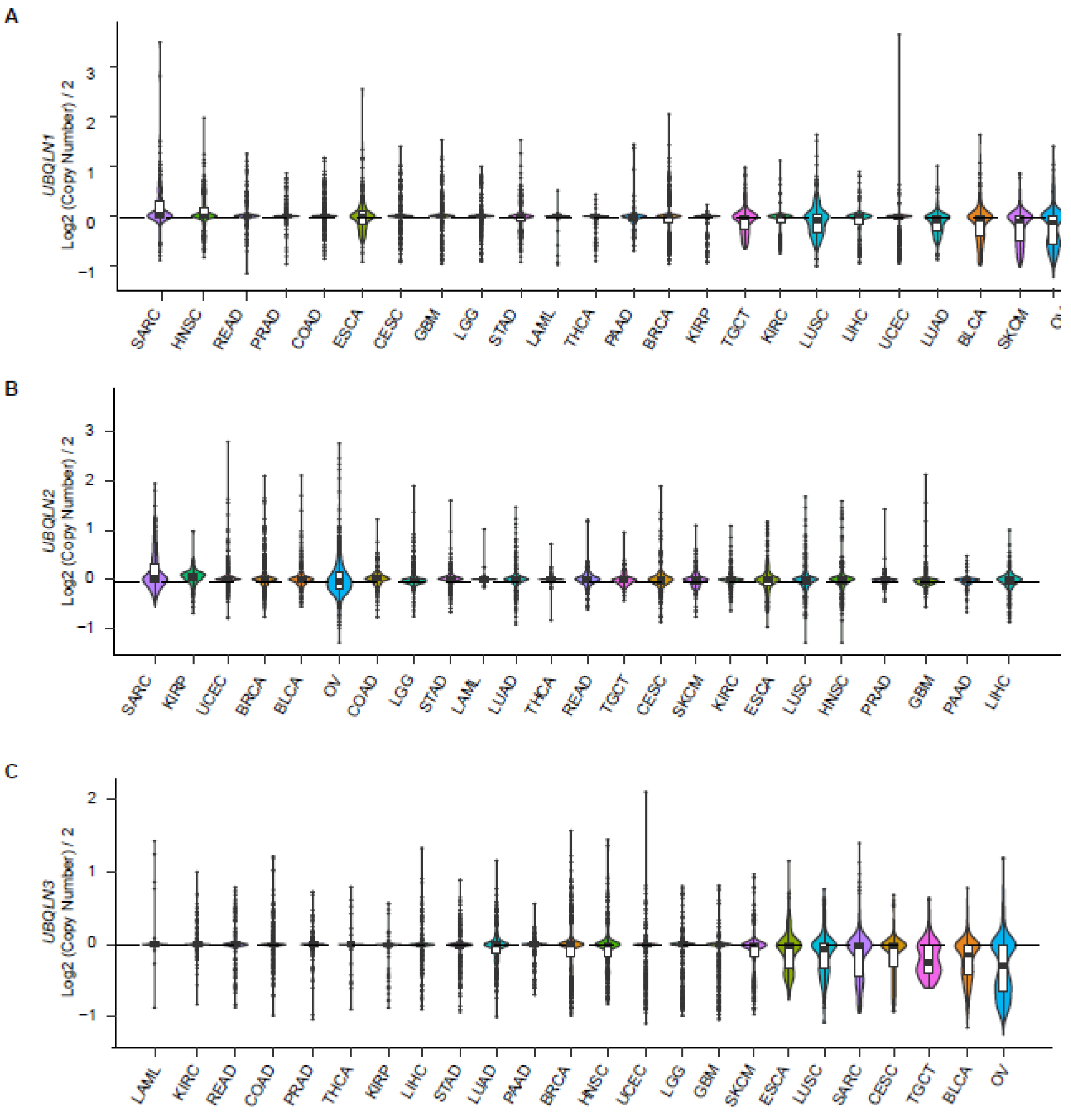

Figure S5. Distributions of DNA copy number of the *UBQLN1-3* genes in different solid tumors in the TCGA database

Distributions of DNA copy number for *UBQLN1* (**A**), *UBQLN2* (**B**), and *UBQLN3* (**C**) genes in different cancer types. Tumor types were sorted by the median value of copy number in individual cancer types.

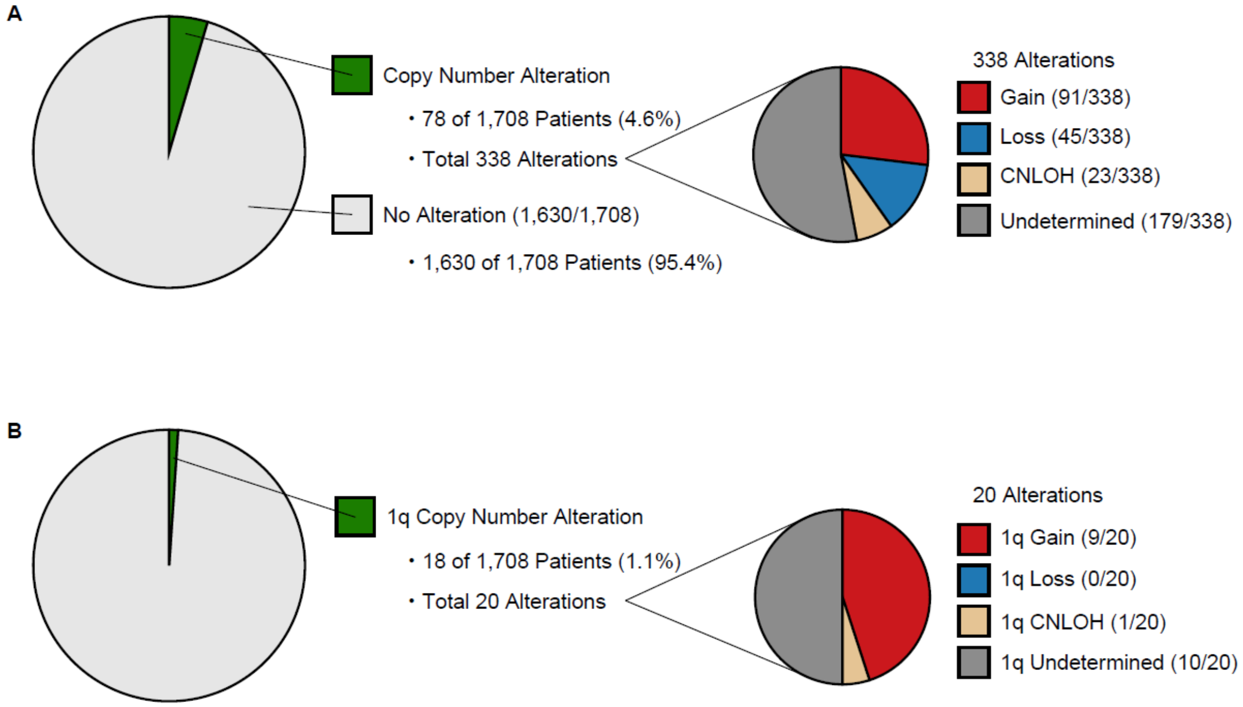

Figure S6. Copy number alterations in adjacent normal tissues in TCGA dataset

A. Pie chart showing the percentage of overall copy number alterations in adjacent normal tissues in TCGA (Left, n = 1,708 patients) and the percentage of each type of alterations (Right, n = 338 alterations). **B.** Pie chart showing the percentage of chromosome 1q copy number alterations in adjacent normal tissues in TCGA (Left, n = 1,708 patients) and the percentage of each type of alterations (Right, n = 20 alterations). CNLOH: copy neutral loss of heterozygosity.

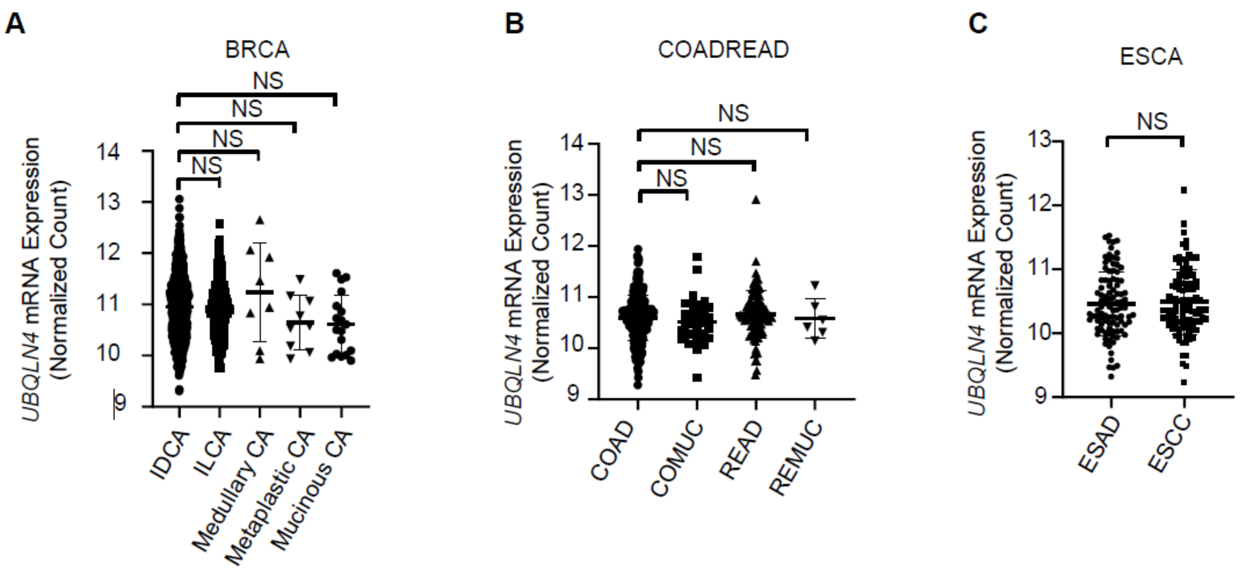

Figure S7. *UBQLN4* mRNA levels in relation to histopathology subtypes in different cancer types

A-C. *UBQLN4* mRNA levels in different histopathology subtypes in different cancer types in TCGA datasets BRCA (A), COAD/READ (B), ESCA (C). COAD: Colon Adenocarcinoma, COMUC: Colon Mucinous Adenocarcinoma, ESAD: Esophageal Adenocarcinoma, ESCC: Esophageal Squamous Cell Carcinoma, READ: Rectal Adenocarcinoma, REMUC: Rectal Mucinous Adenocarcinoma, IDCA: Infiltrating Ductal Carcinoma, ILCA: Infiltrating Lobular Carcinoma, NS; not significant.

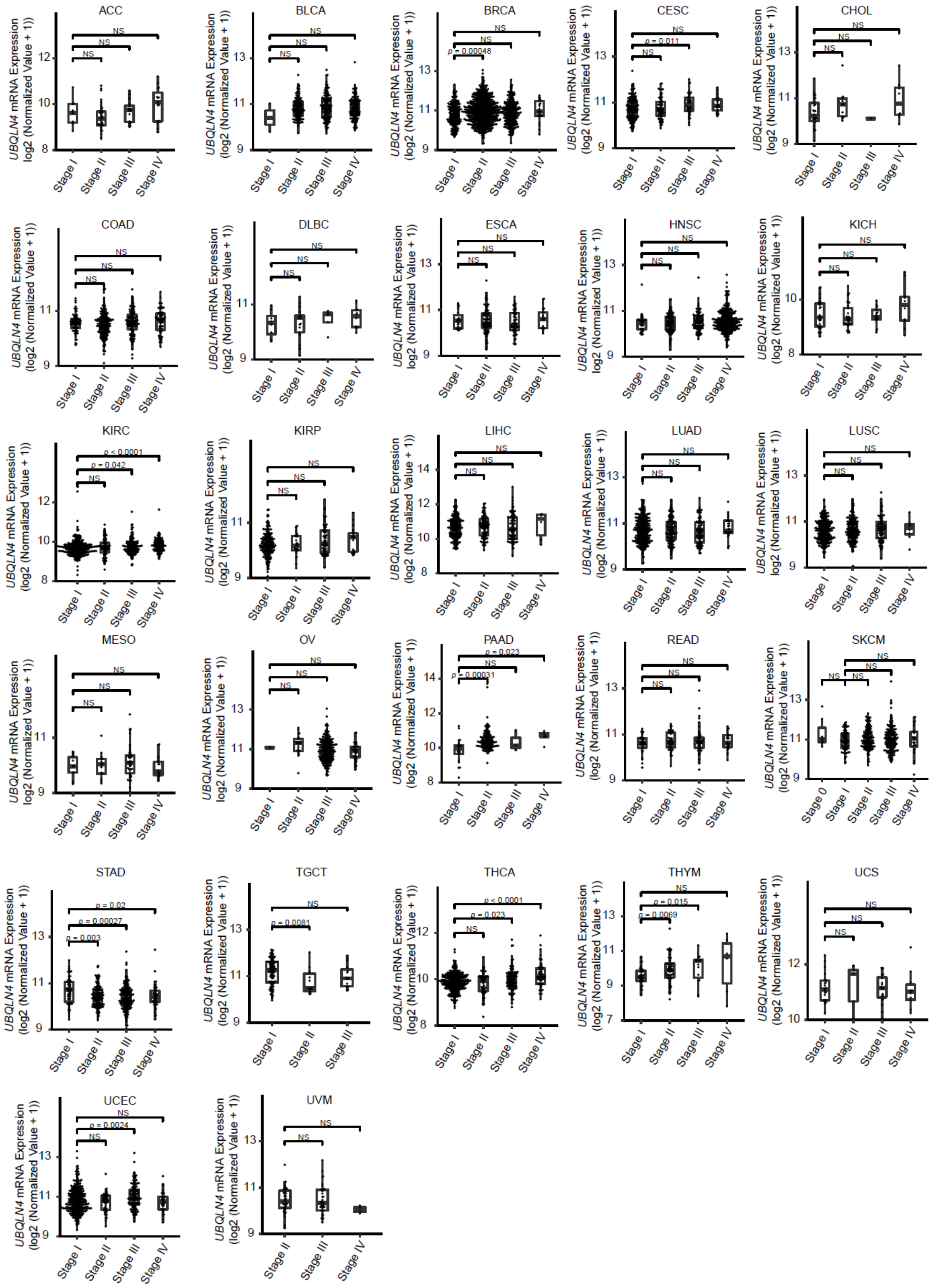

Figure S8. ***UBQLN4*** **mRNA levels in relation to AJCC stage in different cancer types.**

UBQLN4 mRNA levels in cancer patients diagnosed with different stages in the TCGA datasets. ACC: Adrenocortical Carcinoma, BLCA: Bladder Urothelial Carcinoma, BRCA: Breast Invasive Carcinoma, CESC: Cervical Squamous Cell Carcinoma and Endocervical Adenocarcinoma, CHOL: Cholangiocarcinoma, COAD: Colon Adenocarcinoma, DLBC: Lymphoid Neoplasm Diffuse Large B-cell Lymphoma, ESCA: Esophageal Carcinoma, HNSC: Head and Neck Squamous Cell Carcinoma, KICH: Kidney Chromophobe, KIRC: Kidney Renal Clear Cell Carcinoma, KIRP: Kidney Renal Papillary Cell Carcinoma, LIHC: Liver Hepatocellular Carcinoma, LUAD: Lung Adenocarcinoma, LUSC: Lung Squamous Cell Carcinoma, MESO: Mesothelioma, NS: Not Significant, OV: Ovarian Serous Cystadenocarcinoma, PAAD: Pancreatic Adenocarcinoma, READ: Rectal Adenocarcinoma, SKCM: Skin Cutaneous Melanoma, STAD: Stomach Adenocarcinoma, TGCT: Testicular Germ Cell Cancer, THCA: Thyroid Carcinoma, THYM: Thymoma, UCS: Uterine Carcinosarcoma, UCEC: Uterine Corpus Endometrial Carcinoma, UVM: Uveal Melanoma, UCS: Uterine Carcinosarcoma.

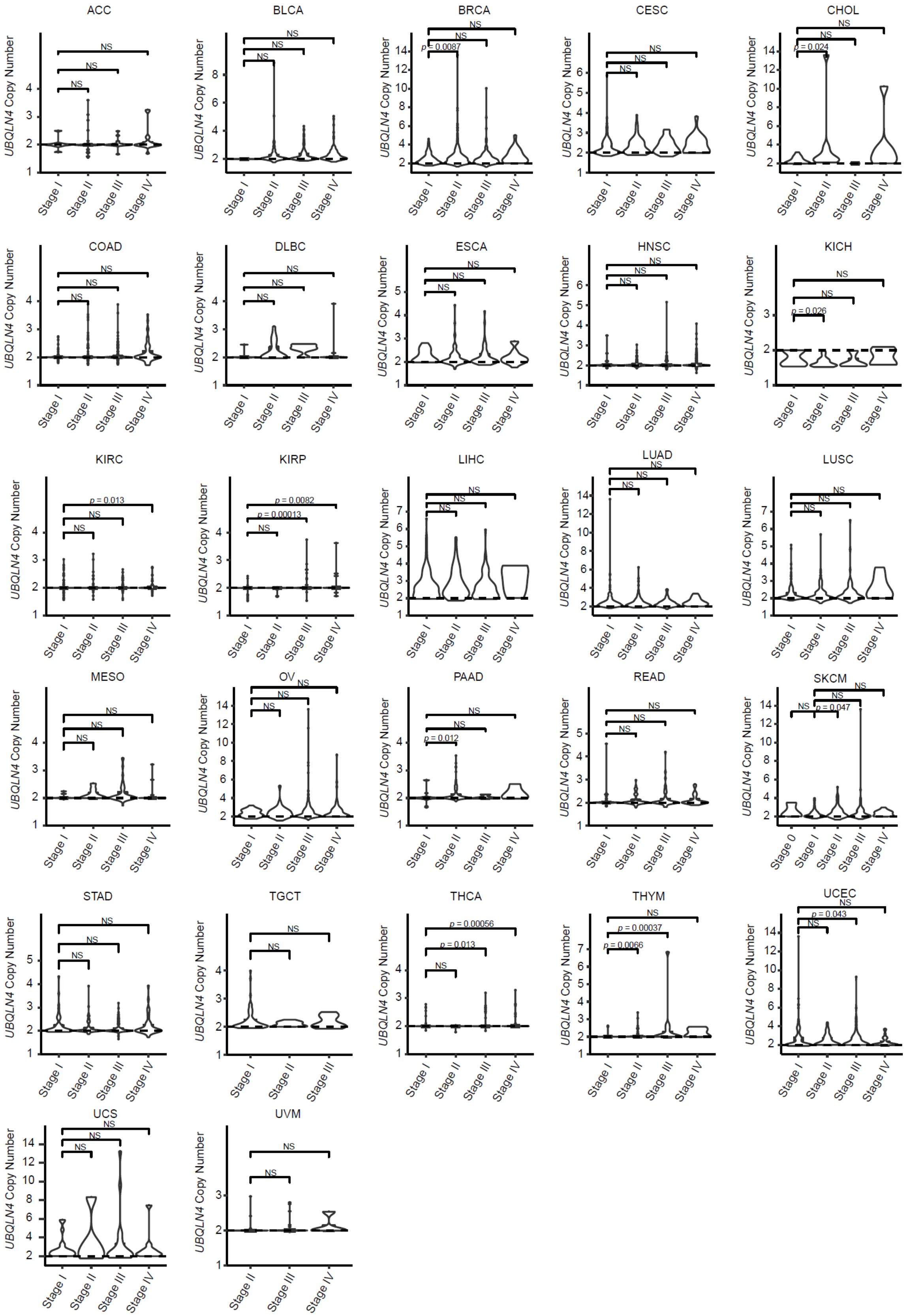

Figure S9. ***UBQLN4*** **DNA copy number in relation to AJCC stages in different cancer types**

UBQLN4 DNA copy number in different stages in different cancer types in TCGA datasets. ACC: Adrenocortical Carcinoma, BLCA: Bladder Urothelial Carcinoma, BRCA: Breast Invasive Carcinoma, CESC: Cervical Squamous Cell Carcinoma and Endocervical Adenocarcinoma, CHOL: Cholangiocarcinoma, COAD: Colon Adenocarcinoma, DLBC: Lymphoid Neoplasm Diffuse Large B-cell Lymphoma, ESCA: Esophageal Carcinoma, HNSC: Head and Neck Squamous Cell Carcinoma, KICH: Kidney Chromophobe, KIRC: Kidney Renal Clear Cell Carcinoma, KIRP: Kidney Renal Papillary Cell Carcinoma, LIHC: Liver Hepatocellular Carcinoma, LUAD: Lung Adenocarcinoma, LUSC: Lung Squamous Cell Carcinoma, MESO: Mesothelioma, NS: Not Significant, OV: Ovarian Serous Cystadenocarcinoma, PAAD: Pancreatic Adenocarcinoma, READ: Rectal Adenocarcinoma, SKCM: Skin Cutaneous Melanoma, STAD: Stomach Adenocarcinoma, TGCT: Testicular Germ Cell Cancer, THCA: Thyroid Carcinoma, THYM: Thymoma, UCS: Uterine Carcinosarcoma, UCEC: Uterine Corpus Endometrial Carcinoma, UVM: Uveal Melanoma.

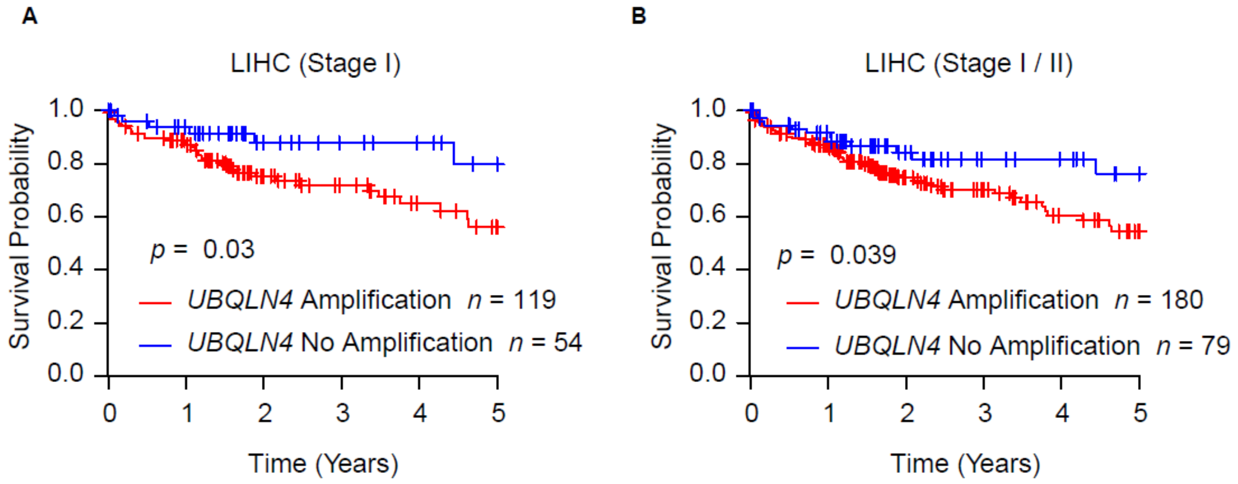

Figure S10. Survival analysis according to *UBQLN4* DNA copy number in Stage I/II patients

A-B. Kaplan-Meier curves for LIHC patients according to *UBQLN4* DNA copy number in Stage I (**A**) and Stage I/II (**B**) of TCGA datasets. Patients with focal *UBQLN4* copy number values larger than 0.3 were defined as *UBQLN4* gene amplification. LIHC: Liver Hepatocellular Carcinoma.

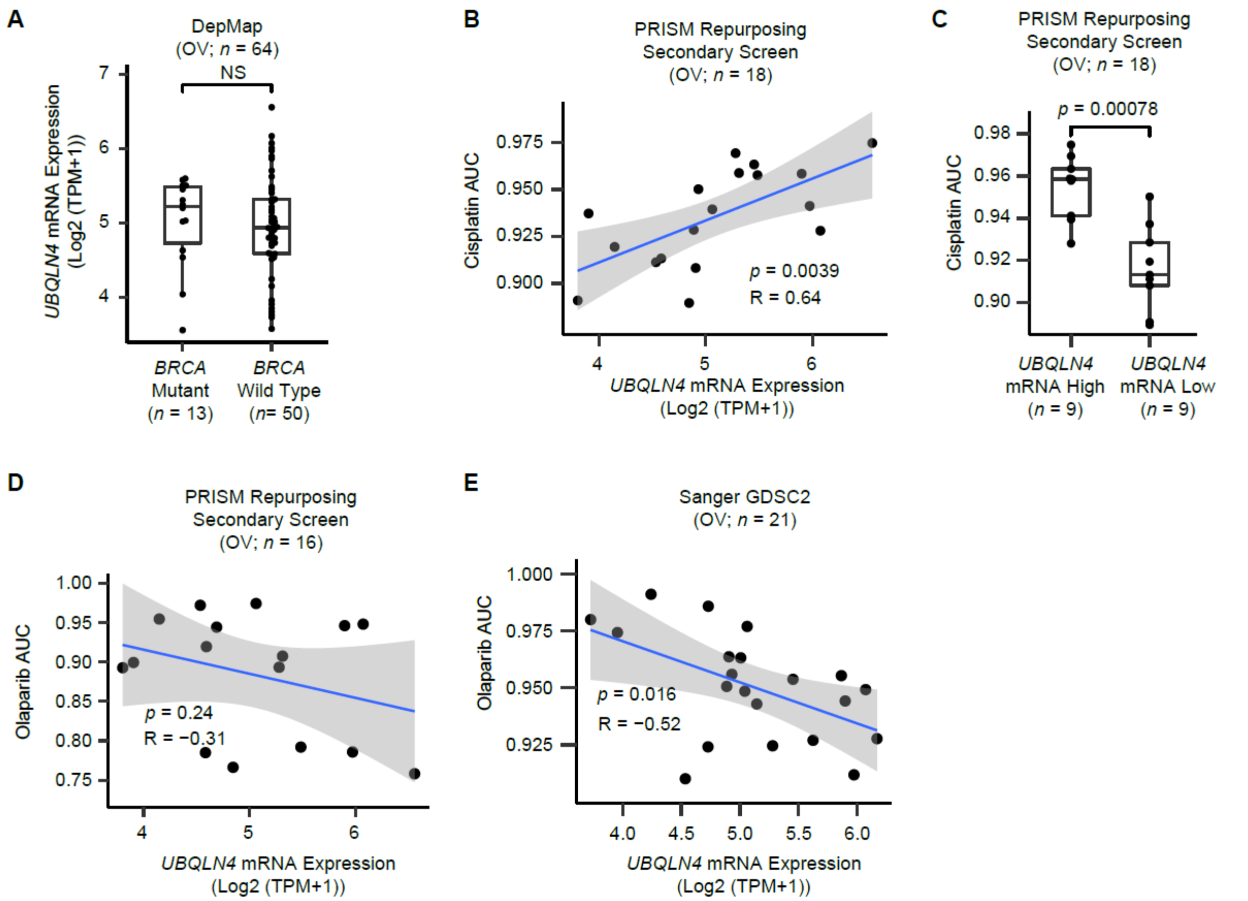

Figure S11. Associations between *UBQLN4* mRNA levels and Cisplatin and Olaparib responses in *BRCA1/2* wild-type ovarian cancer cell lines

A. Comparison of *UBQLN4* mRNA levels in ovarian cancer (OV) cell lines with BRCA mutant and wild-type in DepMap Portal datasets. Statistical difference was evaluated using the Mann Whitney U test. **B.** Pearson's Correlation between *UBQLN4* mRNA levels and cisplatin AUC values in *BRCA1/2* wild-type OV cell lines in PRISM Repurposing Secondary Screen datasets. **C.** Comparison of cisplatin AUC values in *BRCA1/2* wild-type ovarian cancer (OV) cell lines with low and high *UBQLN4* mRNA levels in PRISM Repurposing Secondary Screen datasets. Statistical difference was evaluated using the Mann Whitney U test. **D.** Pearson's correlation between *UBQLN4* mRNA expression and Olaparib AUC values in *BRCA1/2* wild-type OV cell lines in PRISM Repurposing Secondary Screen datasets. **E.** Comparison of Olaparib AUC values in low and high *UBQLN4* mRNA levels cell lines in *BRCA1/2* wild-type OV in Sanger GDSC2 datasets. Statistical difference was evaluated using the Mann Whitney U test. NS: Not Significant.

Table S1. Summary of *UBQLN4* analysis using The Cancer Genome Atlas (TCGA) and The Genotype-Tissue Expression (GTEx)

TCGA ID	Number of tumors in TCGA	Significant difference between TCGA tumor and GTEx normal (p-value***)	Significant difference between TCGA tumor and adjacent normal (p- value***)	*UBQLN4* Amplified Samples (%)	*UBQLN4* Amplification (median CN)	Correlation between *UBQLN4* mRNA and CN*	OS (p-value**)	OS Stage 3 & 4 samples (p-value**)
ACC	79	N/A	N/A	10.98	N (2.00)	0.61	0.0072	0.0055
BLCA	403	Y (= 0.024)	Y (< 0.0001)	33.41	N (2.08)	0.58	0.12	0.14
BRCA	1081	Y (< 0.0001)	Y (< 0.0001)	62.86	Y, High (2.82)	0.66	0.014	0.062
CESC	293	N/A	Y (= 0.021)	42.72	Y, Moderate (2.25)	0.63	0.017	0.28
CHOL	36	N/A	Y (< 0.0001)	61.11	Y, High (2.71)	0.83	0.19	N/A
COAD	447	Y (< 0.0001)	Y (< 0.0001)	14.78	N (2.01)	0.61	0.033	0.14
DLBC	47	N/A	N/A	22.92	N (2.01)	0.54	0.037	0.25
ESCA	184	Y (< 0.0001)	Y (= 0.014)	30.65	Y, Moderate (2.12)	0.64	0.18	0.1
GBM	166	N (< 0.0001)	N (= 0.32)	12.50	N (2.01)	0.28	0.0027	N/A
HNSC	521	N/A	N (= 0.66)	14.52	N (2.01)	0.58	0.016	0.008
KICH	65	N (< 0.0001)	N (< 0.0001)	0	N (1.22)	0.53	0.48	N/A
KIRC	530	N (< 0.0001)	N (< 0.0001)	8.38	N (2.00)	0.45	<0.0001	<0.0001
KIRP	288	N (= 0.062)	Y (< 0.0001)	4.45	N (2.00)	0.47	0.00049	0.088
LGG	525	Y (< 0.0001)	N/A	7.50	N (2.01)	0.13	<0.0001	N/A
LIHC	367	Y (< 0.0001)	Y (< 0.0001)	67.28	Y, High (3.12)	0.63	0.0040	0.056
LUAD	494	Y (< 0.0001)	Y (< 0.0001)	53.55	Y, High (2.54)	0.69	0.011	0.15
LUSC	493	Y (< 0.0001)	Y (< 0.0001)	35.32	Y, Moderate (2.17)	0.6	0.066	0.28
MESO	85	N/A	N/A	20.69	N (2.01)	0.64	0.0012	0.031
OV	307	Y (< 0.0001)	N/A	48.83	Y, High (2.54)	0.66	0.19	0.25

PAAD	178	N (< 0.0001)	N (= 0.2)	19.35	N (2.02)	0.72	0.019	N/A
PCPG	184	N/A	N (= 0.96)	11.96	N (2.02)	0.48	<0.0001	N/A
PRAD	498	N (= 0.06)	Y (< 0.0001)	4.21	N (2.00)	0.55	0.21	N/A
READ	152	Y (< 0.0001)	Y (< 0.0001)	21.56	N (2.01)	0.55	0.22	0.35
SARC	260	N/A	N/A	27.55	N (2.04)	0.64	<0.0001	N/A
SKCM	458	Y (< 0.0001)	N/A	33.33	Y, Moderate (2.24)	0.67	<0.0001	0.00082
STAD	386	Y (< 0.0001)	Y (< 0.0001)	22.12	N (2.02)	0.58	<0.0001[#]	0.00047[#]
TGCT	139	Y (< 0.0001)	N/A	23.74	Y, Moderate (2.10)	0.19	0.65	0.097
THCA	512	N/A	Y (< 0.0001)	4.66	N (2.00)	0.43	0.00055	0.048
THYM	119	N/A	N/A	14.15	N (2.00)	0.50	0.10	N/A
UCEC	530	Y (< 0.0001)	Y (< 0.0001)	42.15	Y, Moderate (2.12)	0.64	<0.0001	0.0016
UCS	55	Y (< 0.0001)	N/A	59.65	Y, High (2.82)	0.77	0.053	0.0069
UVM	80	N/A	N/A	5.29	N (2.00)	0.26	<0.0001[#]	<0.0001[#]

ACC: Adrenocortical Carcinoma, BLCA: Bladder Urothelial Carcinoma, BRCA: Breast Invasive Carcinoma, CESC: Cervical Squamous Cell Carcinoma and Endocervical Adenocarcinoma, CHOL: Cholangiocarcinoma, CN: copy number, COAD: Colon Adenocarcinoma, DLBC: Lymphoid Neoplasm Diffuse Large B-cell Lymphoma, ESCA: Esophageal Carcinoma, GBM: Glioblastoma Multiforme, GTEx: The Genotype-Tissue Expression, HNSC: Head and Neck Squamous Cell Carcinoma, KICH: Kidney Chromophobe, KIRC: Kidney Renal Clear Cell Carcinoma, KIRP: Kidney Renal Papillary Cell Carcinoma, LGG: Low Grade Glioma, LIHC: Liver Hepatocellular Carcinoma, LUAD: Lung Adenocarcinoma, LUSC: Lung Squamous Cell Carcinoma, MESO: Mesothelioma, N: No, N/A: not available, , OS: overall survival, OV: Ovarian Serous Cystadenocarcinoma, PAAD: Pancreatic Adenocarcinoma, PCPG: Pheochromocytoma and Paraganglioma, PRAD: prostate Adenocarcinoma, READ: Rectal

Adenocarcinoma, SARC: Sarcoma, SKCM: Skin Cutaneous Melanoma, STAD: Stomach Adenocarcinoma, TCGA: The Cancer Genome Atlas, TGCT: Testicular Germ Cell Cancer, THCA: Thyroid Carcinoma, THYM: Thymoma, UCEC: Uterine Corpus Endometrial Carcinoma, UCS: Uterine Carcinosarcoma, UVM: Uveal Melanoma. Y: Yes, *: Correlation coefficient calculated by Pearson correlation test, **: *p* value calculated by log-rank test, ***: *p* value calculated by Mann U-Whitney test. [#]: All these *p* -values are associated with OS in patients with low *UBQLN4* mRNA expression.
